# Double aortic arch: a rare cause of adult-onset dysphagia

**DOI:** 10.1055/a-2665-7883

**Published:** 2025-08-08

**Authors:** Xue-Mei Lin, Lin Xia, Xian-Fei Wang, Cong Yuan

**Affiliations:** 1 Department of Pathology, Institute of Basic Medicine and Forensic Medicine, North Sichuan Medical College, Nanchong, China; 2 117913 Department of Pathology, Affiliated Hospital of North Sichuan Medical College, Nanchong, China; 3 117913 Department of Radiology, Affiliated Hospital of North Sichuan Medical College, Nanchong, China; 4 Department of Gastroenterology, Affiliated Hospital of North Sichuan Medical College, Nanchong, China; 5 Digestive Endoscopy Center, Affiliated Hospital of North Sichuan Medical College, Nanchong, China; 6 117913 Department of Gastroenterology, Affiliated Hospital of North Sichuan Medical College, Nanchong, China

A 61-year-old woman complained of intermittent dysphagia to solids for 4 months. There was no previous history of similar episodes, and dyspnea and stridor were also denied. A preliminary esophagogastroduodenoscopy indicated pulsatile subepithelial protrusions in the middle part of the esophagus (22 cm to 30 cm from the incisors), accompanied by significant deformation of the esophageal lumen ( Fig. 1 **a** , Video 1 ). Endoscopic ultrasonography showed that the subepithelial protrusions derived from extraluminal compression rather than intramural lesions ( Fig. 1 **b** ). Contrast-enhanced computed tomography scanning confirmed that the extraluminal compressions were being caused by aberrant vascular configuration, resulting from the double aortic arch (DAA) ( Fig. 2 ). Therefore, it became evident that the patient’s dysphagia was caused by a complete vascular ring due to DAA. The patient refused further surgical intervention.

**Fig. 1:**
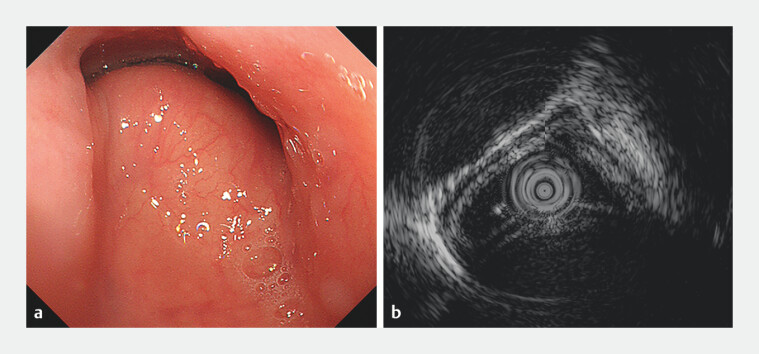
Endoscopic and endosonographic findings of esophageal submucosal protrusions. **a** Endoscopic views showed an esophageal protrusion with normal overlying mucosa 22 cm from the incisors, suggestive of a subepithelial lesion. **b** Endoscopic ultrasonography revealed an intact esophageal wall covering the protrusion, indicating extraluminal compression.

Esophagogastroduodenoscopy indicated subepithelial protrusions and significant luminal deformation in the middle esophagus. Endoscopic ultrasonography and computed tomography confirmed that the changes arose from extraluminal compression by the double aortic arch.Video 1

**Fig. 2:**
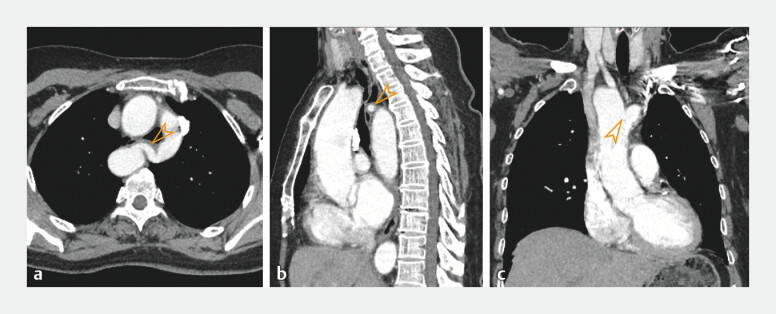
Computed tomography (CT) findings of the chest. Contrast-enhanced CT scanning demonstrated a vascular ring (arrowhead) compressing the esophagus posteriorly on: **a** axial view; **b** sagittal view. The compression resulted in narrowing of the esophageal lumen, corresponding to the esophageal endoscopic views 22 cm away from the incisors. **c** Coronal images showed the ascending aorta bifurcating into the right and left aortic arches (arrowhead).

DAA is a rare congenital cardiovascular condition that results from the failure of the right fourth aortic arch to regress during embryonic development, constituting approximately 1% of cardiovascular congenital anomalies 1 . The vascular rings formed by the DAA may compress the encircled esophagus and trachea, causing dysphagia and wheezing. The clinical manifestations of DAA can differ depending on the degree of tightness of the ring and on subsequent tracheoesophageal compression. Some patients are completely asymptomatic, and others present late in life, as in this case. This patient developed dysphagia later in life, rather than earlier, which may be related to the altered elasticity of the arterial vessels in the elderly 2 .

Endoscopy_UCTN_Code_CCL_1AB_2AC_3AH

## Notes

Endoscopy E-Videos https://eref.thieme.de/e-videos
*E-Videos* is an open access online section of the journal *Endoscopy* , reporting on interesting cases and new techniques in gastroenterological endoscopy. All papers include a high-quality video and are published with a Creative Commons CC-BY license. Endoscopy E-Videos qualify for HINARI discounts and waivers and eligibility is automatically checked during the submission process. We grant 100% waivers to articles whose corresponding authors are based in Group A countries and 50% waivers to those who are based in Group B countries as classified by Research4Life (see: https://www.research4life.org/access/eligibility/ ). This section has its own submission website at https://mc.manuscriptcentral.com/e-videos .
